# A new species of *Monoplectanum sillaginis* sp. nov. (Monopisthocotyla: Diplectanidae) in sillaginid fish from Thailand: the investigations of species diversity, infective situation and morphology

**DOI:** 10.1017/S0031182026101759

**Published:** 2026-04

**Authors:** Nussaba Niyom, Pichit Wiroonpan, Watchariya Purivirojkul

**Affiliations:** 1 Animal Systematics and Ecology Speciality Research Unit, Department of Zoology, Faculty of Science, Kasetsart University, Bangkok, Thailand; 2 Department of Parasitology and Entomology, Faculty of Public Health, Mahidol University, Bangkok, Thailand; 3 Biodiversity Center Kasetsart University (BDCKU), Bangkok, Thailand

**Keywords:** marine parasite, *Monoplectanum*, new species, *Sillago*, The Andaman Sea, The Gulf of Thailand

## Abstract

*Monoplectanum* monogeneans are gill parasites specific to fish in the family Sillaginidae. The sillaginid fish were collected from the middle and upper Gulf of Thailand and the Andaman Sea, Thailand, from May 2024 to January 2025. A total of 2096 fish belonging to 5 species- *Sillago aeolus, S. asiatica S. indica, S. ingenuua* and *S. sihama* were identified. Three species of *Monoplectanum* monogeneans were detected in 3 fish species, including 1 newly described species, *M. sillaginis* sp. nov. *Monoplectanum sillaginis* sp. nov. was found to infect 3 fish species, *S. aeolus, S. indica* and *S. sihama*, collected from the upper Gulf of Thailand. *Monoplectanum youngi* infected *S. indica*, representing a new host record, and *S. sihama* from both the middle and upper Gulf of Thailand. *Monoplectanum australe* was found to infect only *S. aeolus*, which was collected from the upper Gulf of Thailand. Overall prevalence and mean intensity of infections were 11.21% and 1.72, respectively. Regarding morphological features, no significant differences in key characters of both *M. australe* and *M. youngi* were observed when comparing the current and original descriptions. *Monoplectanum sillaginis* sp. nov., can be distinguished from these 2 species by the characteristics of its sclerotized male copulatory organ, particularly in the terminal part of the posterior prostatic reservoir, which is shuttle-shaped, as well as the number of rodlet rows in the squamodisc.

## Graphical abstract

**Figure:**
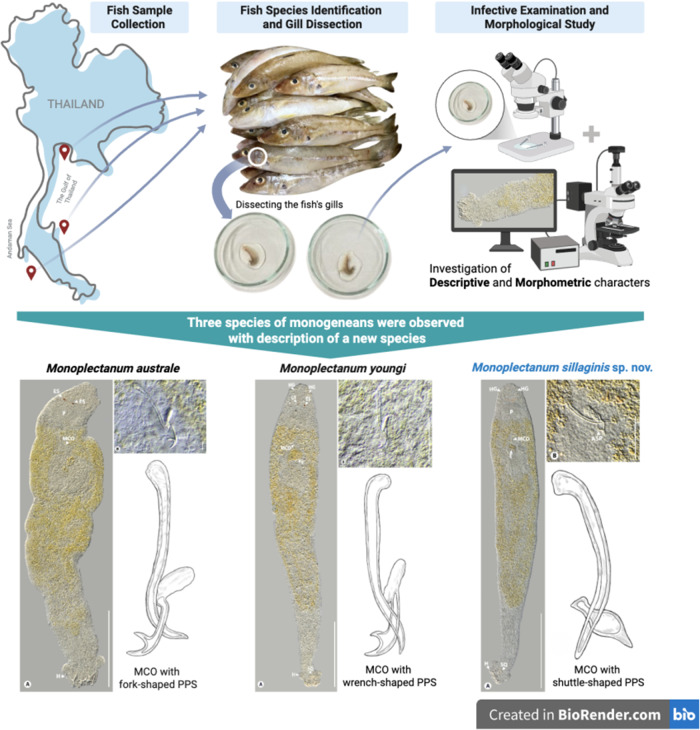

## Introduction

The genus *Monoplectanum* Young 1969 is classified within the family Diplectanidae and is unique by having 1 squamodisc only, whereas other genera typically have 2 (Young, 1969). These monogeneans are ectoparasites found on the gills of fish and, to date, have been reported exclusively from sillaginid fishes. This genus currently includes only 2 previously described species: *M. australe* Young 1969 and *M. youngi* Hayward 1996.

*Monoplectanum australe* infects *Sillago ciliata* Cuvier, 1829, *S. aeolus* Jordan & Evermann, 1902, *S. maculata* Quoy & Gaimard, 1824, and *S. burrus* Richardson, 1842 from Moreton Bay, Shark Bay, Point Samson, Duyfken Point in Australia, and the upper Gulf of Thailand (Young, 1969; Hayward, 1996). However, Hayward (1996), based on Young’s (1969) report, noted that *M. australe* had been incorrectly recorded from *Sillago ciliata*, as no confirmed evidence of infection was found in this host species. Instead, the actual host was likely *S. maculata* (Hayward, 1996). *Monoplectanum youngi* has been reported from several sillaginid fish across a wide geographic range. According to Hayward (1996), this species infects *Sillago* spp. from coastal and estuarine waters of northern and eastern Australia, including Queensland and the Northern Territory, as well as from the Indo-West Pacific region, encompassing Southeast Asia and parts of the western Pacific. Records from Thailand and Malaysia further support its distribution in tropical coastal waters of Southeast Asia.

In this study, we identified 3 parasite species based on the morphology of the sclerotized male copulatory organ, the haptor, and the number of rows in the squamodisc. These species include *M. australe*, which infects *S. aeolus* from both the upper and middle Gulfs of Thailand; *M. youngi*, which infects *S. indica* and *S. sihama* from the upper Gulf of Thailand; and a new species, *M. sillaginis* sp. nov., which infects *S. aeolus, S. indica* and *S. sihama* from the upper Gulf of Thailand. The infective status, morphological description, and distribution of these species are also provided.

## Materials and methods

### Study areas and fish sample collection

A total of 2096 sillaginid fish were collected from the following 3 regions of Thailand: the middle Gulf of Thailand, the upper Gulf of Thailand, and the Andaman Sea. These fish were purchased from fishermen who were able to record and specify the location of their capture. These included 9 localities: SS1 (13°20′45.0″N 100°12′46.0″E), SS2 (13°18′00.4″N 100°08′41.0″E), SS3 (13°12’09.4”N 100°06’21.7”E) in Samut Sakhon Province; ST1 (9°26’56.8”N 100°32’16.9”E), ST2 (10°03’07.7”N 100°00’03.8”E), and ST3 (9°58’50.9”N 100°13’46.5”E) in Surat Thani Province; and T1 (7°06’41.3”N 99°01’46.9”E), T2 (7°11’06.1”N 98°57’26.7”E), and T3 (7°15’23.4”N 98°58’23.1”E) in Trang Province, collected from May 2024 to January 2025. All fish samples were kept on ice and transferred to the laboratory at the Department of Zoology, Faculty of Science, Kasetsart University. Species identification was based on the morphological traits of their swim bladder, following the FAO Species catalogue Vol. 14, Sillaginid Fishes of the World (McKay, 1992).

### Examination of monogenean infection

To collect monogenean parasites, the gills of fish samples were dissected and placed in a Petri dish containing distilled water. The monogenean specimens were then carefully removed from the fish’s gill tissue using a needle under an Olympus SZ51 stereomicroscope (Olympus Corporation, Japan). The collected monogeneans were prepared using 2 methods for morphological investigation. They were mounted in ammonium picrate glycerin for semi-permanent slide preparation to study features such as the haptor, male copulatory organ (MCO), and squamodisc for species identification. For deposition at the museum, the monogeneans were also mounted in Canada balsam to prepare permanent slides. The specimens were photographed with Leica DMi8 and Olympus BX53 microscopes (Olympus Corporation, Japan). Drawings were made with the help of a drawing tube attached to an Olympus BX51 compound light microscope (Olympus Corporation, Japan). Morphological characteristics were measured from specimen images following Chaabane et al. (2017) and Oliva (1968) using ImageJ software (Schneider et al., 2012). The measurements are expressed in micrometers (µm) (Table 2). The monogenean was deposited in the Zoological Museum Kasetsart University as the holotype under accession number ZMKU-PM-2101, 2 paratypes under accession numbers ZMKU-PM-2102 and ZMKU-PM-2103 and 1 paratype was deposited in the Lee Kong Chian Natural History Museum (LKCNHM) at National University of Singapore (NUS) under the following accession number: ZRC.PLA.2258. Infection parameters, including prevalence and mean intensity (MI), were calculated following the definitions given by Margolis et al. (1982) and Bush et al. (1997).

## Results

### Infection rate of *Monoplectanum* spp. in sillaginid fish

**Figure 1.**
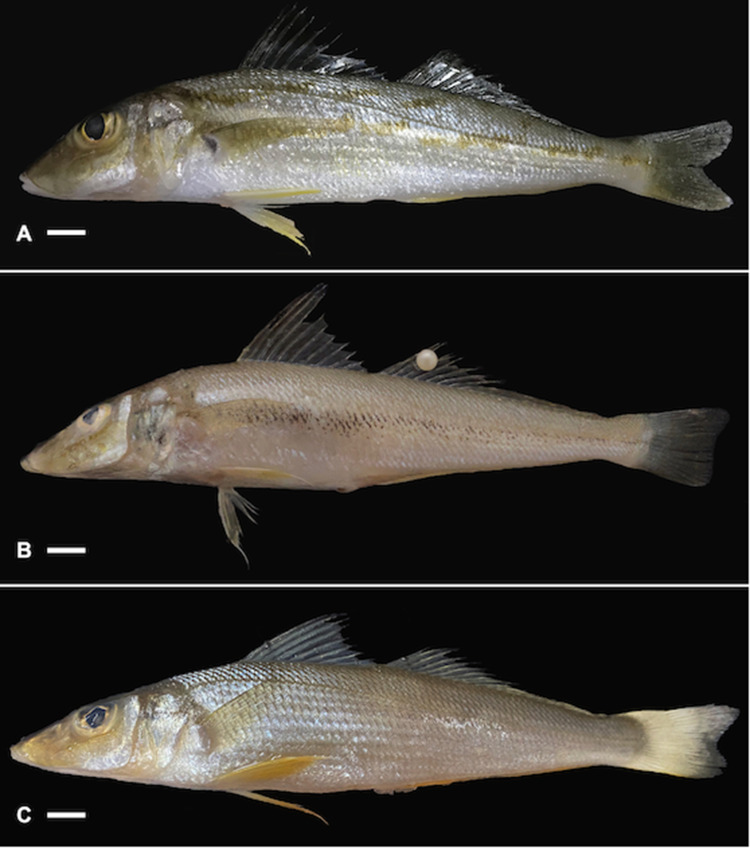
Three sillaginid fish species infected with *Monoplectanum* spp., (A) *S. aeolus*, (B) *S. indica* and (C) *S. sihama*. Scale bars: (A–C) 1 cm. Figure 1 long description.

All 2096 sillaginid fish were examined and classified into 5 species (*S. aeolus, S. asiatica, S. indica, S. ingenuua* and *S. sihama*) (Table 1). Of these, 235 fish belonging to 3 species (Figure 1) of *S. aeolus* (*N* = 105, total length [TL]: 11–26.5 cm), *S. indica* (*N* = 55, TL: 16–28 cm) and *S. sihama* (*N* = 75, TL: 11.7–25 cm) were found to be infected with 3 species of *Monoplectanum* monogeneans, with an overall prevalence of 11.21% and a mean intensity of 1.72 monogeneans per infected fish. The 3 monogenean species were morphologically identified as *M. australe, M. youngi* and *M. sillaginis* sp. nov., the last being described as a new species. Table 1 shows the prevalence and mean intensity of these 3 *Monoplectanum* species infecting the sillaginid fish. *Monoplectanum sillaginis* sp. nov. exhibited the broadest host range, infecting 3 fish species (*S. aeolus, S. indica* and *S. sihama*), followed by *M. youngi*, which was found in 2 species (*S. indica* and *S. sihama*), and *M. australe*, found only in *S. aeolus*. Among the infected sillaginid fish, *S. indica* showed the highest prevalence of monogenean infection at 29.26%, while *S. sihama* had the lowest at 9.18%. Regarding the mean intensity of infection, *S. sihama* displayed the highest at 2.80 (1–7), followed by *S. indica* and *S. aeolus* with 1.63 (1–4) and 1.00, respectively. Among the 3 monogenean species examined, *M. australe* exhibited the highest infective prevalence at 6.79%, while *M. youngi* was the least prevalent at 3.82%. Concerning the mean intensity, *M. youngi* exhibited the highest (2.62).

**Table 1.** Prevalence and mean intensity of the *Monoplectanum* spp. Infections in sillaginid fish Table 1 long description.

		Overall				*M. australe*				*M. youngi*				*M. sillaginis* sp. nov.			
Fish species	No. of examined fish	No. of infected fish	No. of monogenean	Prevalence (%)	Mean intensity	No. of infected fish	No. of monogenean	Prevalence (%)	Mean intensity	No. of infected fish	No. of monogenean	Prevalence (%)	Mean intensity	No. of infected fish	No. of monogenean	Prevalence (%)	Mean intensity
*S. aeolus*	1004	105	105	10.46	1.00	70	70	6.97	1.00	—	—	—	—	35	35	3.49	1.00
*S. asistica*	56	—	—	—	—	—	—	—	—	—	—	—	—	—	—	—	—
*S. indica*	188	55	90	29.26	1.63	—	—	—	—	30	65	15.96	2.17	25	25	13.30	1.00
*S. ingenuua*	60	—	—	—	—	—	—	—	—	—	—	—	—	—	—	—	—
*S. sihama*	788	75	210	9.18	2.80	—	—	—	—	50	145	6.35	2.90	25	65	3.17	2.60
Total	2096	235	405	11.21	1.72	70	70	6.79	1.00	80	210	3.82	2.62	85	85	4.06	1.00

### Infection summary and morphological description of *Monoplectanum* spp

**Figure 2.**
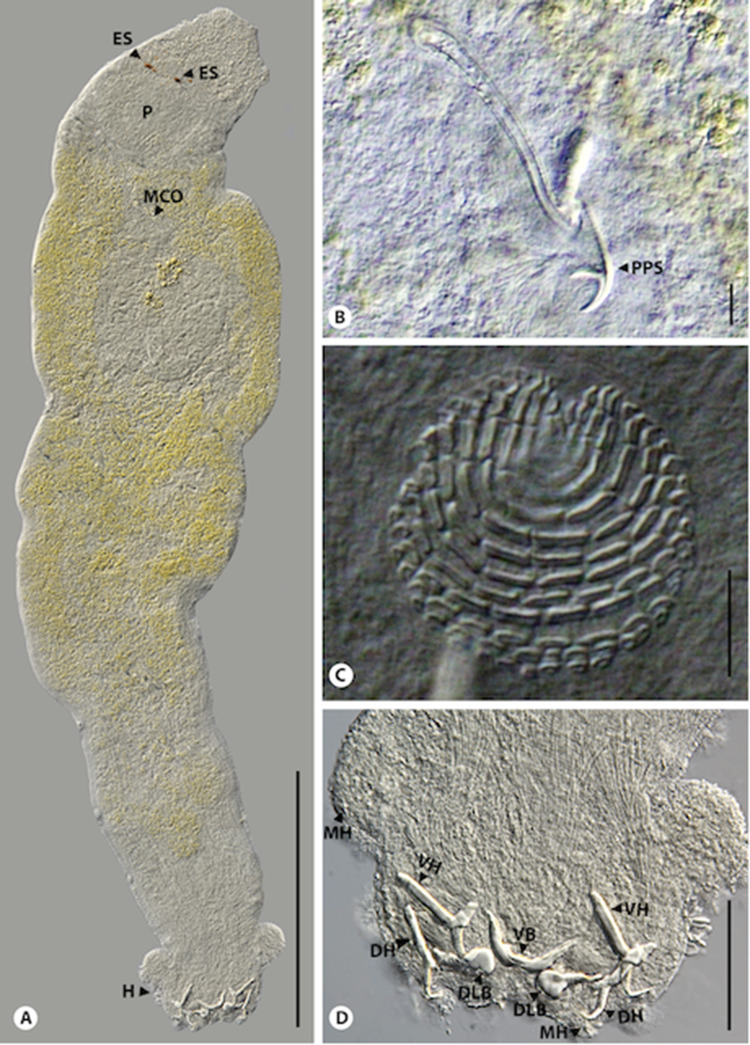
Morphological characters of *Monoplectanum australe* under light microscope. (A) whole body; (B) MCO with posterior prostatic reservoir with fork-shaped; (C) rows of minute rodlets of squamodisc; (D) haptor with hamuli, bar and marginal hooks. Abbreviations as follows: DH: dorsal hamuli; DLB: dorsal (lateral) bar; E: egg; ES: eye spot; H: haptor; HG: Head organ; MCO: male copulatory organ; MH: marginal hook; P: pharynx; PPS: posterior prostatic reservoir; VB: ventral bar; VH: ventral hamuli. Scale bars: (A) 500 µm, (B–C) 10 µm and (D) 50 µm. Figure 2 long description.

***Monoplectanum australe*** Young 1969 (Figure 2)

**Type host**: *Sillago ciliata* Cuvier, 1829

**Present Host**: *Sillago aeolus* Jordan & Evermann, 1902 (Figure 1A)

**Locality**: the upper Gulf of Thailand (13°20’45.0”N 100°12′46.0″E)

**Site of infection**: Gill filaments

**Other records**: *S. ciliata, S. aeolus, S. maculata* and *S. burrus* from Moreton Bay, Shark Bay, Point Samson, Duyfken Point, Australia and the upper Gulf of Thailand, (Young, 1969; Hayward, 1996; Niyom and Purivirojkul, 2020)

### The morphological description

The morphological features of the *M. australe* (Table 2) are based on 10 specimens. The body elongate and slender, 2 pairs of lateral head organs connected to cephalic glands on each side of the pharynx. Two pairs of eye spots: anterior pair smaller than posterior pair. Pharynx spherical. Intestine bifurcates immediately posterior to the pharynx; the caeca and vitellarium terminate well anterior to squamodiscs. Haptor consists of 2 pairs of lateral hamuli, comprised of ventral and dorsal hamuli, 3 bars-1 ventral bar and 2 dorsal (lateral) bars and small squamodisc, along with 14 marginal hooks of the same morphotype.

**Table 2.** Measurement characteristics of 3 species of *Monoplectanum* from the current study and the original description. All measurements are given in micrometers (µm) and abbreviations are as follows: L: Length and W: Width Table 2 long description.

Species	*M. australe* (Present study)	*M. australe* (Young, 1969)	*M. australe* (Hayward, 1996)	*M. youngi* (Present study)	*M. youngi* (Hayward, 1996)	*M. sillaginis* sp.nov. (Present study)
Body (L×W)	1920−2108 (2089) × 341−434 (393)	1152−1600 × 220−288	1420−2450 (1940) × 264−450 (330)	1543−2802 (1917) × 227−384 (311)	1280−1630 (1470) × 226−335 (275)	1511−2101 (1922) × 200−431 (299)
Haptor (L×W)	123−144 (138) × 125−152 (146)	68−92 × 149−176	120−187 (153) (W)	105−157 (131) × 110−207 (150)	97−156 (133) (W)	118−145 (133) × 128−207 (168)
Dorsal hamulus						
Outer length	43−57 (49)	44−48	43−49 (47)	45−67 (49)	37−44 (41)	43−57 (49)
Inner length	18−26 (22)	—	—	20−32 (23)	—	20−28 (23)
Ventral hamulus						
Outer length	40−48 (43)	42−53	42−52 (48)	36−49 (42)	47−51 (49)	38−47 (42)
Inner length	12−19 (15)	—	16−18 (17)	12−19 (15)	16−17 (17)	13−20 (15)
Dorsal lateral bar (L×W)	20−34 (26)× 10−17 (14)	—	26−30 (29) × 12−13 (13)	20−33 (24) × 9−17 (13)	25−30 (28) × 9−12 (10)	21−33 (27) × 9−18 (14)
Ventral bar (L×W)	25−49 (36) × 6−9 (7)	45 × 5−7	—	27−45 (34) × 5−9 (7)	—	29−46 (34) × 5−8 (6)
MCO						
Tube curved (L×W)	60−70 (65) × 4−6 (5)	46−59	56−67 (61) × 4−5 (5)	57−69 (65) × 4−5 (4)	59−68 (62) × 4−5 (5)	60−2 (66) × 4−6 (5)
		3−4				
Posterior prostatic reservoir (L×W)	24−39 (30) (L)	23−28 (L)	—	27−41 (33) (L)	—	23−29 (26) × 7−10 (8)
Squamodisc (L×W)	25−31 (29) × 26−29 (27)	27−35 × 25−37	37−39 (38) × 39 (39)−41(40)	27−30 (29) × 27−32 (29)	33−51 (37) × 24−32 (30)	27−33 (28) × 26−33 (28)
No. of rows of squamodisc	14−16 (15)	15−20	16−17 (16)	14−15 (15)	15−17 (16)	12−13 (13)
Fish hosts	*S. aeolus*	*S. ciliata*	*S. maculata, S. burrus*	*S. indica, S. sihama*	*S. analis, S. attenuata, S. ciliata, S. lutea, S. parvisquamis, S. sihama*	*S. aeolus, S. indica, S. sihama*
Study and distribution areas	Samut Sakhon Province, Thailand	Australia	Australia	Samut Sakhon and Surat Thani Province, Thailand	Australia, Saudi Arabia, Hong Kong, Malaysia and Thailand	Samut Sakhon Province, Thailand

Body elongates, total length (Figure 2A), measuring 1920–2108 (2089) × 341–434 (393). Haptor (Figure 2A, 2B) measures 123–144 (138) × 125–152 (146). Ventral hamuli (Figure 2A, D) have 2 distinct guards, and the tip is recurved, with outer length 40–48 (43) and inner length 12–19 (15). Dorsal hamuli (Figure 2A, D) have an indistinct guard, and the tip is recurved, with outer length 43–57 (49) and inner length 18–26 (22). The single ventral bar bends in a similar V-shape (Figure 2A, D), measuring 25–49 (36) × 6–9 (7). Two dorsal (lateral) bars (Figure 2A, D) are similar in shape, straight, with the initial part flattened, medial end, and articulate laterally with the hamuli (Figure 2A, D), measuring 20–34 (26) × 10–17 (14). Squamodisc consists of rows of minute rodlets, inner row forming closed oral structure (Figure 2C), measuring 25–31 (29) × 26–29 (27), with 14–16 (15) rows connected.

Male copulatory organ (MCO) consists of tube and posterior prostatic reservoir (Figures 2B, 6A). MCO’s tube curved and consists of 2 nested tubes: outer tube surrounding thin-walled inner tube that recurves and thickened proximally, gradually narrowing towards distal end to 60–70 (65) × 4–6 (5). Terminal part of posterior prostatic reservoir fork-shaped (Figures 2B, 6A), measuring 24–39 (30), and posterior prostatic reservoir located at end of tube. Vagina not observed. Vitelline follicles form numerous transversely elongated lobes. Arranged longitudinally along lateral fields of the body, from posterior margin of pharynx to well before position of squamodisc (Figure 2A).

**Remarks**: The species was originally described as *M. australis* by Young (1969), however, Hayward (1996) later referred to it as *M. australe*, which is most likely an erroneous spelling. The specimens examined in this study correspond morphologically to the original description of *M. australe*, particularly in the shape of MCO and haptor. However, this species is very similar to *M. youngi*, such as the number of squamodisc rows, it can be distinguished by differences especially in the shape of MCO and the morphology of the dorsal (lateral) bar.

**Figure 3.**
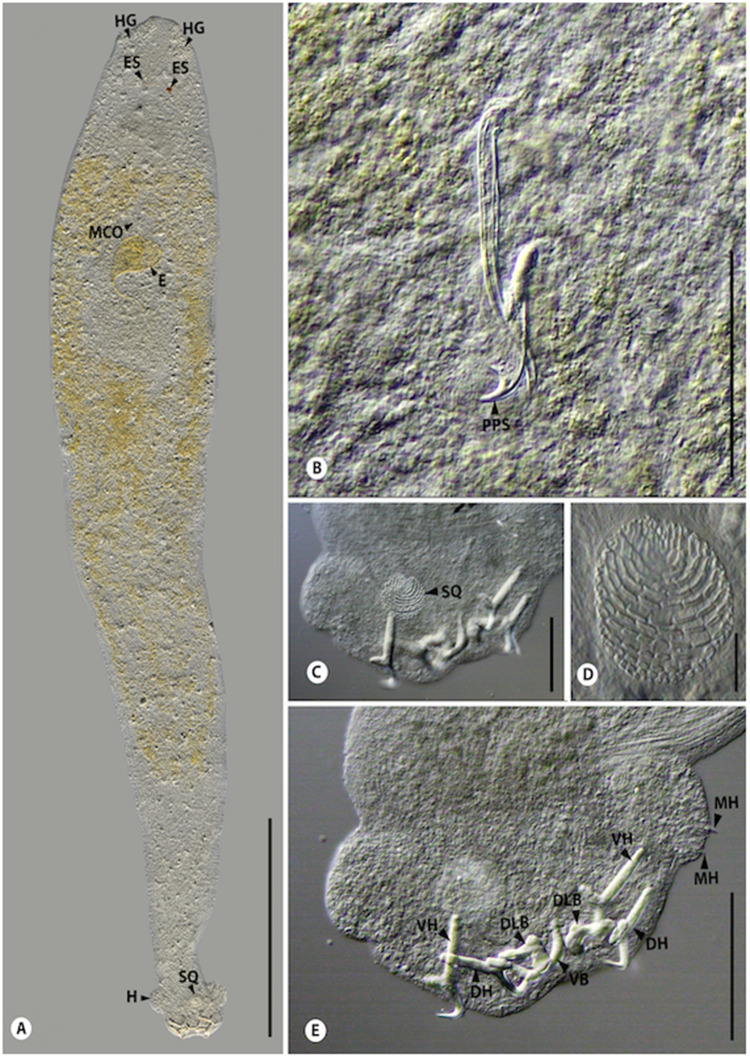
Morphological characters of *Monoplectanum youngi* under light microscope. (A) whole body; (B) MCO with posterior prostatic reservoir with wrench-shaped; (C) position of squamodisc; (D) rows of minute rodlets of squamodisc; (E) haptor with hamuli, bar and marginal hooks. Abbreviations used: DH: dorsal hamuli; DLB: dorsal (lateral) bar; E: egg; ES: eye spot; H: haptor; HG: Head organ; MCO: male copulatory organ; MH; marginal hook; P: pharynx; PPS: posterior prostatic reservoir; SQ: squamodisc; VB: ventral bar; VH: ventral hamuli. Scale bars: (A) 500 µm; (B–C, E) 50 µm and (D) 10 µm. Figure 3 long description.

***Monoplectanum youngi*** Hayward 1996 (Figure 3)

**Type hosts**: *Sillago analis* Whitley, 1943

**Present host**: *Sillago indica* McKay, Dutt & Sujatha, 1985 and *S. sihama* (Forsskål, 1775) (Figure 1B, C)

**Locality**: The upper Gulf of Thailand (13°20′45.0″N 100°12’46.0”E) and the middle Gulf of Thailand (9°26’56.8”N 100°32’16.9”E)

**Site of infection**: Gill Filaments

**Other records**: Gills of *S. analis* (type host), *S. attenuata, S. ciliata, S. lutea, S. parvisquamis* and *S. sihama* from Burdekin River, Point Samson, Princess Charlotte Bay, Torres Strait, Queensland; Moreton Bay, Charles Point, Darwin and Duyfken Point, Australia; Upper Bay Zaal Island, Saudi Arabia; Wan Chai Market, Sai Kung, Hong Kong; Phuket, Bang Saen, Thailand; Kuala Terengganu; and Kuala Lumpur Market, Malaysia (Hayward, 1996).

### The morphological description

The morphological features of the *M. youngi* (Figure 3) are based on 10 specimens. The body elongate and slender, 2 pairs of lateral head organs are connected to cephalic glands on each side of the pharynx. Two pairs of eyespots are present: normally, the anterior pair is smaller than the posterior pair. Pharynx spherical. Intestine bifurcates immediately posterior to pharynx; the caeca and vitellarium terminate well anterior to squamodiscs. Haptor consists of 2 pairs of lateral hamuli, comprising ventral and dorsal hamuli, 3 bars, including 1 ventral bar and 2 dorsal (lateral) bars, and small single squamodisc. There are 14 marginal hooks, all the same morphotype.

Body elongated and slender (Figure 3A), total length, 1543–2802 (1917) × 227–384 (311). Haptor (Figure 3H) measures 105–157 (131) × 110–207 (150). Ventral hamuli (Figure 3E) have 2 distinct guards, and the tip is recurved, with an outer length of 36–49 (42) and an inner length of 12–19 (15). The dorsal hamuli (Figure 3E) have an indistinct guard, and the tip is recurved, with an outer length of 45–67 (49) and an inner length of 20–32 (23). The single ventral bar bends in a similar V-shape (Figure 3H), measuring 27–45 (34) × 5–9 (7). Two dorsal (lateral) bars (Figure 3E) are similar in shape, not overlapping; they are straight and curve at the initial part, expanding wider than the lateral and terminal parts, and articulate with the hamuli laterally. Squamodisc consists of rows of minute rodlets, inner row forming closed oral (Figure 3C, D), measuring 27–30 (29) × 27–33 (29), with 14–15 (15) rows connected.

MCO comprises tube and posterior prostatic reservoir (Figure 3B, 6B). The tube of the MCO curved and consists of 2 nested tubes: outer tube enclosing thin-walled inner tube that recurves, thickened proximally, and gradually narrows along its length towards distal end 57–69 (65) × 4–5 (4). Terminal part of the posterior prostatic reservoir wrench shape (Figures 3B, 6B), 27–41 (33), and the position of the posterior prostatic reservoir is found at the end of the tube. Vagina not observed. Vitelline follicles form numerous transversely elongated lobes. Arranged longitudinally along lateral fields of the body, extending from posterior margin of pharynx to well before position of squamodisc (Figure 3A).

**Remarks**: This species appears to be very closely related to *M. australe*; however, the terminal part of the posterior prostatic reservoir is wrench-shaped, unlike the fork-shaped posterior prostatic reservoir of *M. australe*, and the shape of the dorsal (lateral) bar also differs. It also occurs on various hosts and has a relatively larger distribution than *M. australe*.

**Figure 4.**
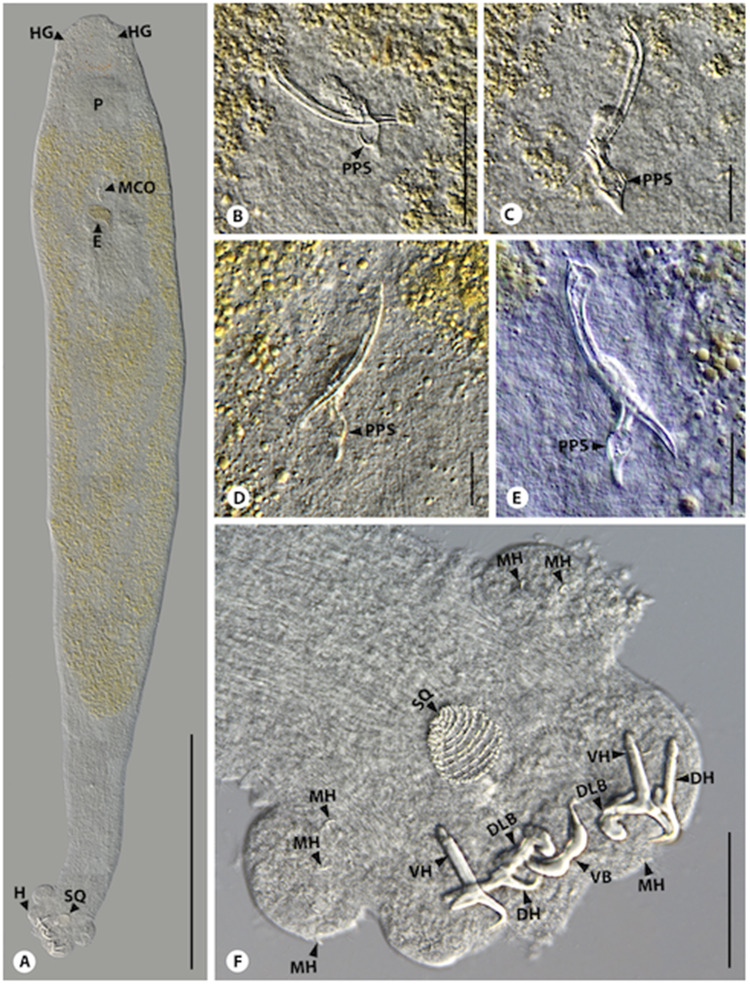
The morphology of *Monoplectanum sillaginis* sp. nov. under light microscope. (A) whole body; (B–E) MCO with different views of posterior prostatic reservoir with shuttle-shaped; (F) haptor with hamuli, bar, marginal hooks and squamodisc. Abbreviations used: DH: dorsal hamuli; DLB: dorsal (lateral) bar; E: egg; ES: eye spot; H: haptor; HG: Head organ; MCO: male copulatory organ; MH: marginal hook; P: pharynx; PPS: posterior prostatic reservoir; SQ: squamodisc; VB: ventral bar; VH: ventral hamuli. Scale bars: (A) 500 µm, (B) 50 µm, (C–E) 20 µm and (F) 50 µm. Figure 4 long description.

**Figure 5.**
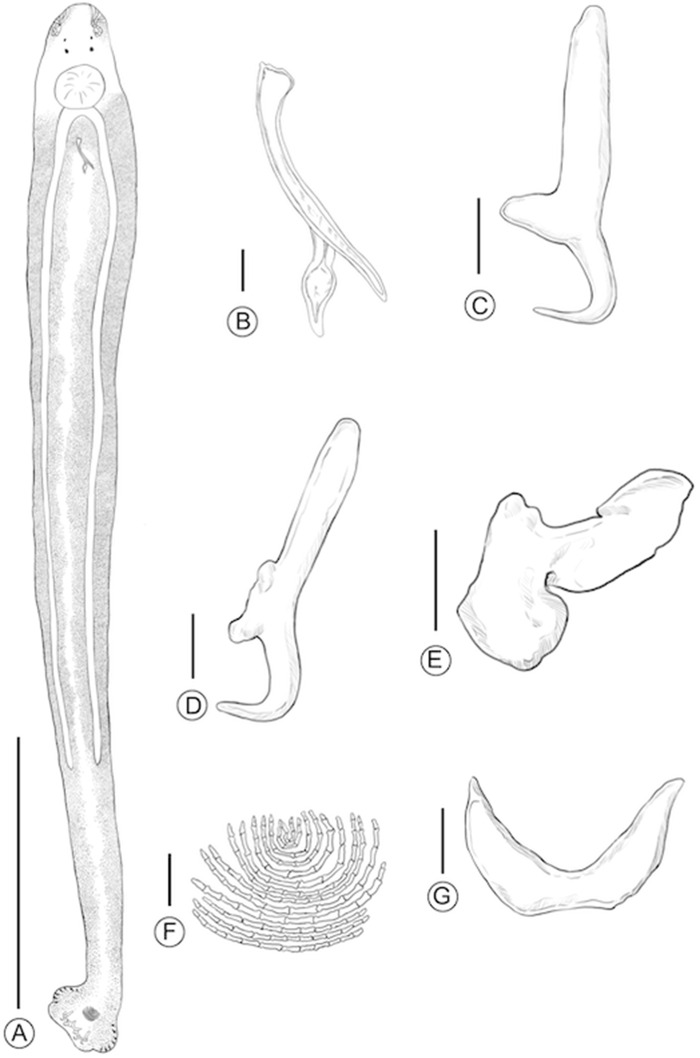
Schematic drawings of *Monoplectanum sillaginis* sp. nov. (A) whole body; (B) MCO; (C) ventral hamuli; (D) dorsal hamuli; (E) dorsal (lateral) bar; (F) squamodisc; (G) ventral bar. Scale bars: (A) 500 µm and (B–G) 10 µm. Figure 5 long description.

***Monoplectanum sillaginis* sp. nov.** (Figures 4, 5)

**Type host**: *Sillago sihama* (Fabricius, 1775) (Figure 1C)

**Other hosts**: *Sillago aeolus* Jordan & Evermann, 1902 and *S. indica* McKay, Dutt & Sujatha, 1985

**Type locality**: Samut Sakhon Province, the upper Gulf of Thailand (13°20′45.0″N 100°12’46.0”E)

**Type specimens**: Holotype, ZMKU-PM-2101; 3 paratypes, ZMKU-PM-2102 and 2103 and ZRC.PLA.2258

**Species LSID**: urn:lsid:zoobank.org:act:47CA1C77-C639-470E-BB2C-F6C8393EA0C0

**Site of infection**: Gill filaments

### The morphological description

The morphological features based on 10 specimens. Slender and elongated body with 2 pairs of lateral head organs connected to cephalic glands on each side of pharynx. There are 2 pairs of eyespots: anterior pair smaller than the posterior pair. Pharynx spherical. Intestine bifurcates immediately posterior to the pharynx; caeca and vitellarium terminate well before the squamodiscs. Haptor consists of 2 pairs of lateral hamuli, including ventral and dorsal hamuli; 3 bars 1 ventral bar and2 dorsal (lateral) bars; small squamodisc; and 14 marginal hooks of the same morphotype.

Body elongated and slender (Figures 4A, 5A), total length 1511–2101 (1922) × 200–431 (299). Haptor (Figure 4A, F) measures 118–145 (133) × 128–207 (168). Ventral hamuli (Figures 4F, 5C) have 2 distinct guards, with the tip recurved; outer length is 38–47 (42), and inner length is 13–20 (15). Dorsal hamuli (Figures 4F, 5D) have an indistinct guard, with the tip recurved; outer length is 43–57 (49), and inner length is 20–28 (23). The single ventral bar is bent into a V-shape (Figures 4F, 5G), measuring 29–46 (34) × 5–8 (6). Two dorsal (lateral) bars (Figures 4F, 5E) are similar in shape, not overlapping; they are straight and curve at the initial part, widening more than the lateral and terminal parts, and articulate with the hamuli laterally. The squamodisc consists of rows of minute rodlets and an inner row forming a closed oral structure (Figures 4A, F, 5F), measuring 27–33 (28) × 26–33 (28), with 12–14 (13) rows connected.

**Figure 6.**
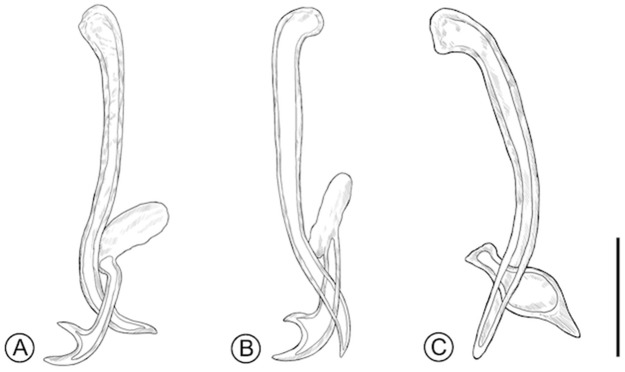
Schematic drawings of MCO differences of 3 *Monoplectanum* species, (A) *M. australe* (fork-shaped); (B) *M. youngi* (wrench-shaped); (C) *M. sillaginis* sp. nov. (shuttle-shaped). Scale bar: 20 µm. Figure 6 long description.

MCO comprises the tube and posterior prostatic reservoir (Figures 4B–E, 5B, 6A–E). Tube of the MCO is curved and consists of two nested tubes: an outer tube encases a thin-walled inner tube that is recurved and thickened proximally, gradually narrowing along its length toward the distal end 60–72 (66) × 4–6 (5). Terminal part of posterior prostatic reservoir shuttle-shaped (Figures 4B–E, 5B, 6C), measuring 23–29 (26) × 7–10 (8). Posterior prostatic reservoir positioned two-thirds along the length of the tube. Vagina not observed. Vitelline follicles form numerous transversely elongated lobes. Arranged longitudinally along lateral fields of the body from posterior margin of pharynx to just before squamodisc (Figure 4A, 5A).

**Differential diagnosis**: *Monoplectanum sillaginis* sp. nov. is clearly distinguished from other *Monoplectanum* species by the morphology of the MCO and the number of rodlet rows in the squamodisc. In *M. sillaginis* sp. nov., the terminal part of the posterior prostatic reservoir of the MCO is shuttle-shaped, and the squamodisc comprises 12–13 (13) rows of rodlets. In contrast, *M. australe* possesses a fork-shaped terminal part of posterior prostatic reservoir and squamodisc with 14–16 (15) rows, whereas *M. youngi* is characterized by a wrench-shaped terminal part of posterior prostatic reservoir and squamodisc bearing 14–15 (15) rows.

## Discussion

Sillaginid fish are widely distributed in the Indo-Pacific region, including Thailand, especially the Andaman Sea and the Gulf of Thailand (McKay, 1992; Froese and Pauly, 2025). However, the investigation of their parasitic monogenean infections in Thailand is still limited. Among 5 sillaginid fish species collected in the current study, 3 of them were observed to harbour *Monoplectanum* spp. Among 3 species of monogeneans currently observed, *M. sillaginis* sp. nov. shows distinct morphological differences from other species in the genus *Monoplectanum*, especially in the structure of the MCO and the number of rodlet rows in the squamodisc.

The general morphology of the 2 *Monoplectanum* species, *M. australe* and *M. youngi*, as observed in our study, agrees with the original description by Hayward (1996) regarding body shape, organ appearance, and the position and arrangement of organs. However, the measurements of *M. australe* in our study showed that at least 2 organs, the ventral hamulus and squamodisc, were smaller than those reported in the original description (Table 2). For *M. youngi*, an organ of the ventral hamulus was found to be smaller than previously reported (Table 2). These differences in morphological features based on measurements may be related to specimen preservation and preparation processes, as well as the measurement methods used (Justine, 2005; Fankoua et al., 2017).

Our results show the prevalence and mean intensity of infection by 3 *Monoplectanum* species in 3 sillaginid fishes (Table 1). The overall prevalence and mean intensity of the infection were 11.21% and 1.72 monogeneans per infected fish, respectively. *Monoplectanum australe* was found only in *S. aeolus*, with a prevalence and mean intensity of 6.97% and 1, respectively, meanwhile no infection was found in the remaining fish species. Compared to a previous study (Niyom and Purivirojkul, 2020) that they reported *M. australe* infecting *S. aeolus* fish with 2.82% and 3 of prevalence and mean intensity of the infection, the present study shows a higher prevalence and lower mean intensity that reported *M. australe* in 2 fish species, including *S. maculata* with high infection (20%, 2) in comparison to *S. aeolus* with a low infection (2.82%, 3), the present study shows a higher prevalence and lower mean intensity. Variations in sampling years, environmental conditions, or host-parasite interactions may cause this difference. Infection of the *M. sillaginis* sp. nov. observed in 3 fish species might suggest high infectability of them compared to the remaining examined monogeneans. Moreover, infection of different hosts can reduce the risk of extinction due to fluctuations in host abundance (Tavares-Dias et al., 2022). However, some previous studies did not provide detailed data about the infection rates, making it to be difficult to compare the infective prevalence and mean intensity with earlier findings.

**Figure 7.**
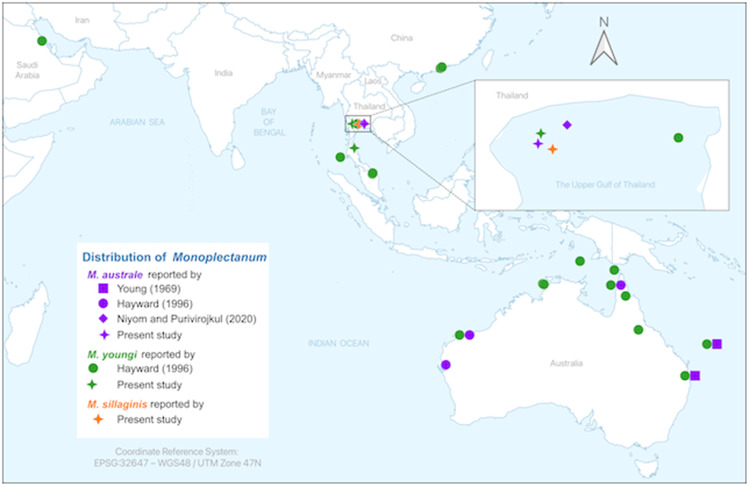
Distribution map of *Monoplectanum* spp. reported by previous and current studies. Different colours indicate different *Monoplectanum* species, and different symbol shapes denote different sources of study. This map was created using QGIS version 3.34 based on the following coordinate reference system: ESPG:32647–WGS48/UTM Zone 47N (QGIS Development Team, 2021). Figure 7 long description.

Distribution of the *Monoplectanum* has been primarily reported in the Oriental and Australian faunal regions (Figure 7), from 1969 to the presence (Young, 1969; Hayward, 1996; Niyom and Purivirojkul, 2020). Species of *Monoplectanum* are distributed according to the dispersion of sillaginid fish, with several species serving as hosts for *Monoplectanum* spp. (Hayward, 1996; Niyom and Purivirojkul, 2020). Different species of *Monoplectanum* have varying host ranges, despite being distributed in the same region, especially in Australia. Between the 2 species, the distribution range of *M. youngi* is broader than that of *M. australe. Monoplectanum youngi* has been recorded in several areas of Australia, Malaysia, Thailand, Hong Kong, and even Saudi Arabia, while *M. australe* is known to be distributed in Australia and Thailand. The difference in the distribution ranges of these 2 monogeneans may be due to the taxon spectrum and distribution ability of their fish hosts. Seven species of the following sillaginid fish have been documented as hosts of *M. youngi: S. analis, S. attenuata, S. ciliata, S. indica, S. lutea, S. parvisquamis* and *S. sihama*, while 4 sillaginid species (*S. aeolus, S. burru, S. ciliata* and *S. maculata*) have harboured *M. australe*. This broader spectrum of host species undoubtedly contributes to the enhancement of parasite distribution. One of these sillaginid species, *S. sihama* (host of *M. youngi*), exhibits a distribution range throughout the Indo-West Pacific region (from South Africa to Japan) (McKay, 1992; Froese and Pauly, 2025), demonstrating that its wider distribution could also expand the range of *M. youngi*. Furthermore, the distribution ability of *M. youngi* may relate to its size, particularly its body size. Compared to *M. australe, M. youngi* has a smaller body size, based on both this study’s comparative data and previous research. The smaller size of the monogenean may reduce the chance of dislodging from the gill filament in water currents, leading to a broader distribution following its fish host. Regarding the new species, *M. sillaginis* sp. nov., it is currently reported to be distributed from the upper Gulf of Thailand and infects 3 sillaginid species, 2 of which (*S. aeolus* and *S. sihama*) have wider distributions across the oriental and Australian regions (McKay, 1992; Froese and Pauly, 2025). Consequently, the distribution of *M. sillaginis* sp. nov. could potentially expand similarly to *M. youngi* due to their shared fish hosts (*S. sihama* and *S. indica*), especially *S. sihama*, which is distributed throughout the Indo-West Pacific region (McKay, 1992; Froese and Pauly, 2025). Focusing on Thailand, the upper Gulf region currently shows the distribution of all 3 *Monoplectanum* species. Meanwhile, the middle Gulf of Thailand, also documented for *M. youngi* in this study.

In conclusion, our investigation identified 3 species of *Monoplectanum* from the upper and the middle Gulf of Thailand, including *M. australe, M. youngi* and *M. sillaginis* sp. nov., the latter of which was described as a new species. Key characteristics distinguishing these 3 species were clearly defined and demonstrated. The infection data revealed that *M. sillaginis* sp. nov. was the most prevalent species infecting sillaginid fish. Additionally, the study provided information on the distribution of monogeneans. The data obtained may contribute to a better understanding of the biodiversity of monogeneans in marine ecosystems. While this study primarily focused on morphological characteristics, future research should consider incorporating ecological approaches and molecular character to clarify infection patterns or dynamics of the monogeneans and their fish host related to environmental factors.

## Figure 1 Long description

The image shows three sillaginid fish species in lateral view, labeled A, B and C. Each fish is displayed horizontally with visible fins and scales. The fish labeled A has a streamlined body with distinct dorsal and pectoral fins. The fish labeled B appears slightly elongated with visible dorsal and anal fins. The fish labeled C has a prominent dorsal fin and a tapered tail. Scale bars are present beneath each fish, indicating size reference.

Navigate back to Figure 1.

## Figure 2 Long description

Image A shows the whole body of Monoplectanum australe with labeled parts: eye spot, pharynx, male copulatory organ and haptor. Image B displays the male copulatory organ with a fork-shaped posterior prostatic reservoir. Image C presents rows of minute rodlets of the squamodisc. Image D illustrates the haptor with hamuli, bar and marginal hooks, including dorsal hamuli, ventral bar, ventral hamuli and marginal hook. Scale bars are visible in each image, indicating magnification levels.

Navigate back to Figure 2.

## Figure 3 Long description

Image A shows the whole body of Monoplectanum youngi with labeled parts: eye spot, head organ, male copulatory organ, egg, pharynx, haptor and squamodisc. Image B displays the male copulatory organ with a posterior prostatic reservoir, wrench-shaped. Image C illustrates the position of the squamodisc. Image D shows rows of minute rodlets of the squamodisc. Image E depicts the haptor with dorsal hamuli, ventral hamuli, dorsal lateral bar, ventral bar and marginal hooks. Scale bars are visible in each image, indicating magnification levels.

Navigate back to Figure 3.

## Figure 4 Long description

The image A shows the whole body of Monoplectanum sillaginis sp. nov. labeled with HG subscript a, HG, P, MCO, E and SQ. The image B to E show different views of the posterior prostatic reservoir labeled as PPS. The image F shows the haptor with various structures labeled as MH, VH, DH, DLB, VB and SQ. The haptor includes hamuli, bar, marginal hooks and squamodisc. Scale bars are visible in each image, indicating the magnification level.

Navigate back to Figure 4.

## Figure 5 Long description

The image shows schematic drawings of Monoplectanum sillaginis sp. nov. A depicts the whole body with a scale bar of 500 micrometers. B shows the MCO with a scale bar of 10 micrometers. C illustrates the ventral hamuli, also with a 10 micrometer scale bar. D presents the dorsal hamuli, accompanied by a 10 micrometer scale bar. E displays the dorsal (lateral) bar with a 10 micrometer scale bar. F features the squamodisc and G shows the ventral bar, both with 10 micrometer scale bars.

Navigate back to Figure 5.

## Figure 6 Long description

The image shows three schematic drawings labeled A, B and C, depicting male copulatory organs with different shapes. Drawing A illustrates a fork-shaped organ, characterized by a bifurcated structure at the base. Drawing B presents a wrench-shaped organ, featuring a curved design with a prominent hook-like element. Drawing C shows a shuttle-shaped organ, distinguished by a streamlined form with a rounded end. A scale bar is visible next to the drawings, indicating size reference.

Navigate back to Figure 6.

## Figure 7 Long description

The map illustrates the distribution of Monoplectanum species across various regions, including Australia, Thailand, Malaysia and Saudi Arabia. Different symbols represent sources of study: triangles for Young (1969), squares for Hayward (1996), diamonds for Niyom and Purivirojkul (2020) and circles for the present study. The species M. australe, M. youngi and M. sillaginis are marked with different colors. An inset map highlights the upper Gulf of Thailand, showing detailed distribution points. The map uses the coordinate reference system ESPG:32647–WGS48/UTM Zone 47N.

Navigate back to Figure 7.

## Table 1 Long description

The table measures the prevalence and mean intensity of Monoplectanum spp. infections in various sillaginid fish species. S. indica has the highest prevalence at 29.26%, with a mean intensity of 1.63, indicating frequent infections. S. aeolus and S. sihama also show notable infection rates, with prevalences of 10.46% and 9.18%, respectively. S. asistica and S. ingenuua have no recorded infections, suggesting possible resistance or absence of the parasite. The data highlights that M. australe primarily infects S. aeolus, while M. youngi and M. sillaginis sp. nov. are more prevalent in S. indica and S. sihama. Overall, the total prevalence across all species is 11.21%, with varying mean intensities, indicating differences in infection severity among species.

Navigate back to Table 1.

## Table 2 Long description

The table compares measurement characteristics of three Monoplectanum species across different studies and regions, focusing on body and haptor dimensions, hamulus lengths, and other anatomical features. M. australe from the present study has the largest body length and width compared to previous studies by Young and Hayward. M. youngi shows a wide range of body sizes, with the present study reporting larger dimensions than Hayward's 1996 study. M. sillaginis, a new species, has measurements similar to M. youngi. The dorsal and ventral hamulus lengths are consistent across species, with slight variations. Fish hosts and study locations vary, with M. australe and M. sillaginis found in Thailand, while M. youngi is distributed across multiple countries. These variations highlight the influence of geographical and environmental factors on species morphology.

Navigate back to Table 2.
